# Lactate: creatinine ratio in babies with thin meconium staining of amniotic fluid

**DOI:** 10.1186/1471-2431-6-13

**Published:** 2006-04-20

**Authors:** Rishi Kant Ojha, Saroj K Singh, Sanjay Batra, V Sreenivas, Jacob M Puliyel

**Affiliations:** 1 Department of Pediatrics and Neonatology, St. Stephen's Hospital, Tis Hazari, New Delhi, India; 2 Department of Biochemistry, Kalawati Saran Children's Hospital, Connaught place, New Delhi, India; 3 Department of Biostatistics, All India Institute of Medical Sciences, Ansari Nagar, New Delhi, India

## Abstract

**Background:**

ACOG states meconium stained amniotic fluid (MSAF) as one of the historical indicators of perinatal asphyxia. Thick meconium along with other indicators is used to identify babies with severe intrapartum asphyxia. Lactate creatinine ratio (L: C ratio) of 0.64 or higher in first passed urine of babies suffering severe intrapartum asphyxia has been shown to predict Hypoxic Ischaemic Encephalopathy (HIE). Literature review shows that meconium is passed in distress and thin meconium results from mixing and dilution over time, which may be hours to days. Thin meconium may thus be used as an indicator of antepartum asphyxia. We tested L: C ratios in a group of babies born through thin and thick meconium, and for comparison, in a group of babies without meconium at birth.

**Methods:**

86 consecutive newborns, 36 to 42 weeks of gestation, with meconium staining of liquor, were recruited for the study. 52 voided urine within 6 hours of birth; of these 27 had thick meconium and 25 had thin meconium at birth. 42 others, who did not have meconium or any other signs of asphyxia at birth provided controls. Lactate and creatinine levels in urine were tested by standard enzymatic methods in the three groups.

**Results:**

Lactate values are highest in the thin MSAF group followed by the thick MSAF and controls. Creatinine was lowest in the thin MSAF, followed by thick MSAF and controls. Normal babies had an average L: C ratio of 0.13 (± 0.09). L: C ratio was more among thin MSAF babies (4.3 ± 11.94) than thick MSAF babies (0.35 ± 0.35). Median L: C ratio was also higher in the thin MSAF group. Variation in the values of these parameters is observed to be high in the thin MSAF group as compared to other groups. L: C ratio was above the cutoff of 0.64 of Huang et al in 40% of those with thin meconium. 2 of these developed signs of HIE with convulsions (HIE Sarnat and Sarnat Stage II) during hospital stay. One had L: C Ratio of 93 and the other of 58.6. A smaller proportion (20%) of those with thick meconium had levels above the cutoff and 2 developed HIE and convulsions with L: C ratio of 1.25 and 1.1 respectively.

**Conclusion:**

In evolving a cutoff of L: C ratios that would be highly sensitive and specific (0.64), Huang et al studied it in a series of babies with severe intrapartum asphyxia. Our study shows that the specificity may not be as good if babies born through thin meconium are also included. L: C ratios are much higher in babies with thin meconium. It may be that meconium alone is not a good indicator of asphyxia and the risk of HIE. However, if the presence of meconium implies asphyxia then perhaps a higher cut-off than 0.64 is needed. L: C ratios should be tested in a larger sample that includes babies with thin meconium, before L: C ratios can be applied universally.

## Background

Anoxic injury to the fetal brain as a result of birth asphyxia may lead to cerebral palsy. Intrapartum asphyxia alone however accounts for only a small proportion of cases of cerebral palsy (CP) [1,2]. In a consensus statement, the Australian and New Zealand Perinatal Societies have estimated that around 10% of cases of cerebral palsy stem from adverse intrapartum events [3]. In the majority, the cause of CP is antepartum [4]. Intrinsic fetal causes like intracranial malformations and inborn errors of metabolism contribute to a miniscule number of cases.

The American College of Obstetricians and Gynecologists (ACOG) has laid down compulsory criteria, all of which must be met for establishing a plausible link between *intrapartum asphyxia *and neurological deficit. These are – profound acidaemia, Apgar score of 3 or less for more than 5 minutes, neonatal neurological sequelae (like seizures), and multiorgan system dysfunction [5].

The manifestations of *antepartum asphyxia *are poorly understood and most are not specifically related to brain damage. Reduced fetal movements, non-reassuring fetal heart rate patterns [6](FHRT), fetal ECG [7], fetal scalp blood sampling [8] and in-utero passage of meconium [9] are some of them. Lack of specificity of these indicators limits their use in medicolegal issues. Epidemiologically, as most damage in cases of cerebral palsy is antepartum, each of these 'nonspecific' indicators need careful scrutiny to identify antepartum asphyxia.

As with intrapartum asphyxia, most babies with antepartum asphyxia escape damage. In only a few babies with asphyxia is there neonatal encephalopathy. Some of these may progress to Hypoxic ischaemic encephalopathy (HIE), and only a subset of those with HIE eventually sustain brain damage [10].

12%–22% of all pregnancies are complicated by meconium, but 50% of those who develop HIE had been born through meconium stained amniotic fluid [11](MSAF). Meconium in labor may indicate fetal distress or asphyxia. There are at least three theories explaining meconium passage: in-utero relaxation of the anal sphincter [12] vagal stimulation [12,13] and postmaturity [14]. Numerous investigators have concluded that presence of meconium in the amniotic fluid is a sign of fetal hypoxia or acidosis [15-19].

Recent studies [20,21] have reported much higher levels of fetal erythropoetin, and lower fetal oxygen saturation [22] in pregnancies complicated by meconium and concluded that in-utero passage of meconium reflects chronic asphyxia. In summary, it is understood that, (though not and independent predictor of poor neurodevelopmental outcome), intrauterine passage of meconium may reflect an asphyxial event.

Some babies pass meconium due to intrauterine infections, chorioamnionitis, and premature rupture of membranes and up to35% of postmature babies (born after 42 weeks of gestation) may pass meconium [23]. Clearly, asphyxia cannot alone be implicated for in-utero meconium passage especially in post-mature babies and in the presence of intrauterine infections.

When freshly passed, meconium is a thick, viscous, green liquid [24] except perhaps in cases of fetal diarrhea due to Listeriosis. Meconium stains the amniotic fluid and presents as thick meconium. The consistency is altered over time by dilution and fetal movement [25] resulting in thin meconium. Arguably, thinner and more uniformly mixed meconium has been retained in the amniotic fluid for a longer duration (hours to days)[25], than meconium that appears thick. The exact time interval between passage of meconium to its complete dilution is not available in literature.

After excluding postmature babies and those who are likely to be infected, we assume for purposes of this study that all babies who pass meconium have asphyxia. Those who pass thick meconium are assumed to have intrapartum asphyxia and those with thin meconium to have antepartum asphyxia. We tested the premise with Lactate: Creatinine ratio (L: C ratio)- a proven marker of asphyxia.

Combining lactate- a product of anaerobic metabolism resulting from *hypoxia*, and creatinine-a measure of renal function whose excretion is reduced in renal compromise caused by *ischaemia*, L: C ratio in urine has been shown to predict HIE and future neurodevelopmental outcome. Huang et al [26] studied a group of severely asphyxiated newborns, identified by well-established criteria of perinatal asphyxia (thick meconium among others) and reported that L: C ratio of 0.64 or higher in first passed urine was highly specific for HIE [5].

We used L: C ratio as a marker to study antepartum and intrapartum asphyxia. We tested L: C ratio in a group of term babies, born to mothers without a history of intrauterine infection, through thin and thick meconium, and for comparison in a group of normal babies without meconium.

## Methods

The study was conducted at St Stephen's Hospital, Delhi India. It is a 600 bedded tertiary level postgraduate teaching hospital, conducting 5000 deliveries annually, equipped with a 75 bedded nursery and level III neonatal intensive care unit.

**Figure 1:**
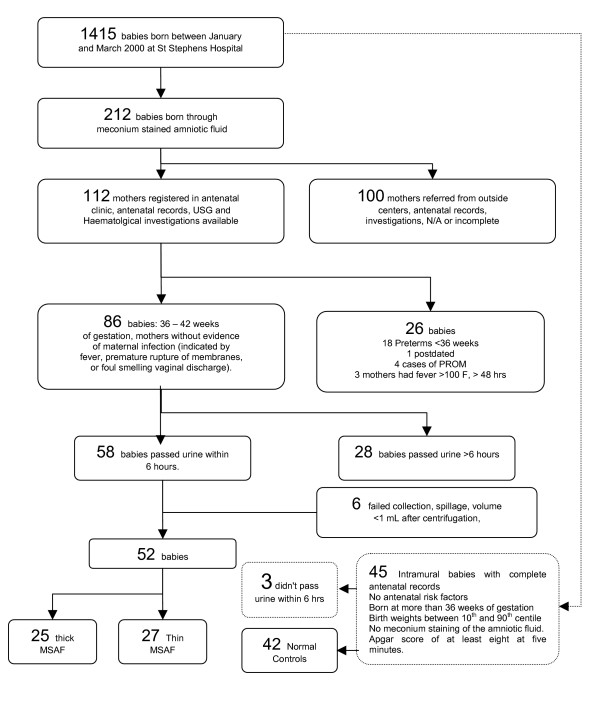
Schematic representation of the study population to show how the cases were recruited for the study.

Of 1415 deliveries between January to March 2000 at our hospital, 212 babies were born through MSAF. Some of these mothers were referred from other centers in labor and antenatal records were not available. These mothers were excluded. The number of eligible mothers was thus 112. From among these, post term mothers and mothers with a history suggestive of infection were excluded. 86 consecutive newborns of 36–42 weeks of gestation with meconium staining of amniotic fluid, without evidence of maternal infection (indicated by fever, premature rupture of membranes, or foul smelling vaginal discharge), were thus subjects of the study. None of the babies had any renal abnormality, or other congenital anomalies detected at antenatal ultrasonographic examination. 28 babies did not pass urine within six hours. Urine from 6 babies could not be analyzed as volume available, after centrifugation (to remove the heavy precipitates in neonatal urine), storage, and transportation to the laboratory was found inadequate. Finally more than 1 ml of clear supernatant urine passed within six hours of birth was available for testing from 52 babies and this group of samples was analyzed. Figure 1 shows a flow diagram of how the cases were recruited.

Meconium was categorized into thick or thin, based on its naked-eye appearance, by the pediatrician or nurse attending the delivery. Among the babies whose urine was available, 27 had had thick meconium and 25 had thin meconium. For comparison, we collected samples of urine, passed within six hours of birth from 42 normal babies born at more than 36 weeks of gestation with no staining of the amniotic fluid. These babies had Apgar score of at least eight at five minutes. This was essentially a 'convenience sample' collected from babies of consenting parents who met the above criteria to act as controls.

The pediatric unit looked after all babies during their hospital stay. Blood gas analysis was done when indicated as per hospital protocol. For staging of HIE, the classification of Sarnat and Sarnat [28] was used. The score uses clinical parameters like heart rate, respiration, pupils, consciousness, muscle tone, posture and neonatal reflexes and electroencephalographic (EEG) criteria, as well as incidence of seizures not attributable to transient metabolic derangements. Whereas a detailed clinical scoring, and incidence of seizures was recorded in all suspected babies, EEG was not done. The hospital research committee approved the study. Informed consent was obtained from parents of babies participating in the study, prior to urine collection.

Samples of the first voided urine were centrifuged and clear supernatant was transferred to 1-ml plain Greiner cones (Greiner Labortechnik GmbH, Maybachstra, Frickenhausen, Germany) and preserved at minus 32°C in a deep freezer. All samples were tested together for urine lactate and creatinine by conventional enzymatic method by a Randox™ kit (Randox Laboratories, Co. Antrim, UK) on Olympus (Reply) autoanalyzer (Olympus Optical Co. Ltd, Shinjuku-ku, Tokyo, Japan).

The oxidase enzyme in the kit converts lactate to pyruvic acid and hydrogen peroxide, which results in oxidative conversion of chromogen to produce a coloured dye with absorption maximum at 540 nm, the amount of which is directly proportional to the amount of lactate in the sample. Creatinine in urine was measured by the concentration of a yellow orange colored dye formed from the reaction with Picric acid under alkaline conditions, which had its absorption maximum at 500 nm.

The continuous and quantitative variables like birth weight, gestational age were compared among the three groups of subjects, namely normal babies, those with thin and thick MSAF, using the analysis of variance technique. Lactate, creatinine and L: C ratio values were analyzed with ANOVA after logarithmic transformation followed by Bonferroni adjustment for multiple comparisons. The Bonferroni correction is part of the 'one-way' command of Stata ver 8.0, which gives the actual p values for each specific comparison. It, like any other multiple comparison procedure, is relevant only when one finds a significant difference among the groups being compared by ANOVA. Bonferroni correction is a more conservative procedure than other multiple comparison procedures. Thus, if Bonferroni correction leads to a significant result, we can be more confident of the significance of the result.

Qualitative variables like sex, Apgar score (categorization into two groups – <6 at 5 minutes and ≥ 6 at 5 minutes), maternal and postnatal problems (again categorized-present or absent) were compared among the three groups by Fisher's exact test. All the statistical analyses were carried out using STATA **8.0**. p – value of less than 0.05 was considered to be statistically significant.

## Results

Table 1 shows the population characteristics of the three groups of babies: those born with thin MSAF, those with thick MSAF and normal babies. They were comparable in terms of gestational age, gender distribution, and Apgar scores at 5 minutes. Problems during gestation like pregnancy induced hypertension, cephalopelvic disproportion, and fetal heart rate abnormalities, were associated more with thick meconium stained babies compared to controls (p < 0.01) than with thin meconium compared to controls. Four mothers in the control group had antenatal problems. Two mothers had gestational diabetes (on diet control), along with hypertensive (on treatment), one had hypertension alone (on treatment) and one mother had leaking of nearly 20 hours duration. Normal babies on the average weighed 120 grams more than babies with thin MSAF and 230 grams more than babies with thick MSAF. However, only thick MSAF babies weighed significantly lower compared to controls (Bonferroni corrected p = 0.02). Difference in the birth weights between thin and thick MSAF babies was not statistically significant (Bonferroni corrected p = 0.69).

**Table 1:** Characteristics of newborns in the study: 27 newborns with thin MSAF, 25 newborns with thick MSAF and 42 controls

**Characteristic**	**Normal infants**	**Infants with MSAF (n = 52)**		
	(n = 42)	Thin (n = 27)	Thick (n = 25)	**p- Value^$^**
**Birth weight (**±**SD)**	2885.7 ± 374 g	2767.0 ± 289.9 g	2654.8 ± 311 g^@^	**0.03**
				
**Gestational Age (**±**SD)**	40 Wks ± 1.57 Wk	39 Wks ± 2.0 Wks	39 Wks ± 2.0 Wks	**0.96**
				
**Sex**				
*Male*	18 (42.9%)	19 (70.4%)	13 (52.0%)	
*Female*	24 (57.1%)	8 (29.6%)	12 (48.0%)	**0.08**
				
**Apgar Score**				
*>6 at 5 min*	42 (100.0%)	25 (92.5%)	22 (88.0%)	
*<6 AT 5 min*	0 (0.0%)	2 (7.4%)	3 (12.0%)	**0.07**
				
**Maternal problems**				
*None*	38 (90.5%)	25 (92.6%)	13 (52.0%)	
Significant maternal problems*	4 (10.0%)	2 (7.4%)	12 (48.0%)^@^	**<0.01**
				
**Mode of Delivery**				
*Normal vaginal delivery*	39 (92.9%)	18 (66.7%)	16 (64.0%)	
*Forceps Delivery*	3 (7.1%)	4 (14.8%)	3 (12.0%)	
*Caesarian section*	0 (0.0%)	5 (18.5%)	6 (24.0%)^@^	**<0.01**
				
**Postnatal problems**				
*None*	42 (100%)	23 (85.2%)	18 (72.0%)	
Other problems**	0 (0.0%)	4 (14.8%)	7 (28.0%)^@^	0.001
				
**Hypoxic Ischaemic Encephalopathy (HIE)**				
No	42 (100%)	25 (92.6%)	23 (92.0%)	
Yes	0 (0.0)%	2 (7.4%)	2 (8.0%)	0.12

*Thick MSAF babies had significantly more antenatal problems – 3 cases of maternal hypertension, 2 cases of gestational diabetes, 3 cases of fetal heart rate abnormality (FHRT), 2 cases of breech delivery, and one case each of cephalopelvic disproportion, and intrauterine growth retardation. Thin MSAF Babies had fewer problems in the mothers with two cases of hypertension of which one mother also had cephalopelvic disproportion.

**2 children born through Thin MSAF developed Group B Streptococcal septicaemia,2 developed respiratory distress attributable to aspiration pneumonia, and one child developed hyperbilirubinaemia requiring phototherapy. In the Thick MSAF Group, 4 developed respiratory distress attributable to aspiration pneumonia, 2 developed hyperbilirubinaemia requiring phototherapy and one child had congenital heart disease.

$ Based on One-Way ANOVA/Fisher's Exact test

@Significant difference of thick MSAF group in comparison to controls.

Thick MSAF babies had three cases of maternal hypertension, 3 cases of fetal heart rate abnormality (FHRT), two cases of breech delivery, and one case each of cephalopelvic disproportion, and intrauterine growth retardation. Thin MSAF Babies had fewer problems in the mothers with two cases of hypertension and one case of cephalopelvic disproportion. Two babies born through thick MSAF who developed HIE had history of maternal hypertension, neither had FHRT and both were delivered vaginally, and had low apgar scores. The two babies born through thin MSAF who developed HIE were without any maternal hypertension, no FHRT, and hence both were delivered vaginally. Apgar score of one was 3 at 5 minutes but the other had an Apgar score of 8, developed neuromotor excitability and excessive crying and later developed seizures. All controls were born vaginally, 24% (n = 6) with thick MSAF and 18% (n = 5) with thin were born through Caesarian section.

Postnatally,2 babies born through thin MSAF developed Group B Streptococcal septicaemia,2 developed respiratory distress attributable to aspiration pneumonia, and one child developed hyperbilirubinaemia requiring phototherapy. In the Thick MSAF Group, 4 developed respiratory distress attributable to aspiration pneumonia, 2 developed hyperbilirubinaemia requiring phototherapy and one child had congenital heart disease. None of the babies with HIE had any congenital malformation detected at antenatal ultrasonography, clinical examination at birth or postnatal cranial ultrasonography.

Table 2 compares the values of lactate, creatinine and L: C ratios in babies with thin MSAF, thick MSAF and normal babies. Lactate values are highest in the thin MSAF group followed by the thick MSAF and controls. Creatinine, on the other hand, was lowest in the thin MSAF, followed by thick MSAF and controls. Normal babies had an average L: C ratio of 0.13 (± 0.09). It can be noted that the L: C ratio was more among thin MSAF babies (4.3 ± 11.94) than thick MSAF babies (0.35 ± 0.35). Median L:C ratios were also higher in the thin MSAF group. Variation in the values of these parameters is observed to be high in the thin MSAF group as compared to other groups.

**Table 2:** Urine Lactate, Creatinine and L: C Ratio in first passed urine within six hours of birth from normal babies, those with thick and thin meconium staining of liquor at birth. (p-values comparing the means by ANOVA on log transformed values

	**Controls **(n = 42)	**Thin MSAF **(n = 27)	**Thick MSAF **(n = 25)	**P value^$^**
**Lactate (mg/dL)**				
Mean ± SD	3.3 ± 2.79^@^	21.6 ± 34.83	5.1 ± 5.34	**0.02**
Median (IQR)	1.8 (1.30 – 5.05)	6.7 (1.10 – 14.60)	3.5 (1.49 – 6.40)	

**Creatinine (mg/dL)**				
Mean ± SD	25.3 ± 10.08^@^	17.7 ± 13.32	20.4 ± 13.71	**< 0.01**
Median (IQR)	26.2 (19.58 – 32.00)	15.7 (7.00 – 25.70)	20.3 (8.50 – 30.80)	

**L:C Ratio**				
Mean ± SD	0.13 ± 0.09^@^	4.33 ± 11.94	0.35 ± 0.35	**< 0.01**
Median (IQR)	0.09 (0.06 – 0.18)	0.29 (0.09 – 1.11)	0.23 (0.11 – 0.48)	

$ Based on One-way ANOVA

^@^: Significant difference between Controls and Thin MSAF (on Bonferroni adjusted multiple comparisons).

Though there are significant differences in the means of the three parameters among the three groups, multiple comparison procedures showed the difference as significant only when normal babies were compared to babies with thin MSAF (Bonferroni corrected p values: lactate 0.02, creatinine 0.01, LC ratio < 0.001). In other words, differences between thin and thick MSAF groups are not significant (Bonferroni corrected p values: lactate 0.34, Creatinine 0.75, LC ratio 0.09) and also that between thick MSAF and control groups (Bonferroni corrected p values: lactate 0.90, creatinine: 0.26, LC ratio 0.14). There was no significant difference between thick MSAF and controls.

Median values of the three parameters also show similar pattern in the three groups.

**Figure 2:**
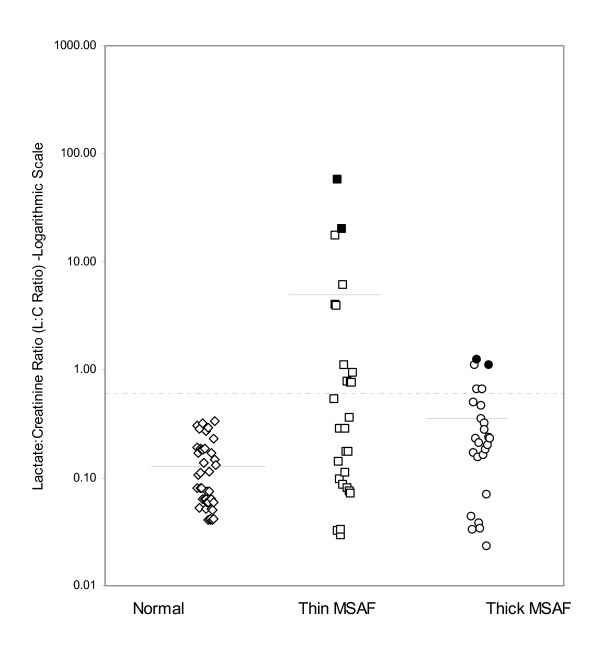
Lactate: Creatinine ratio (L:C ratio) in the normal population compared against thin and thick meconium stained babies, logarithmic scale. The cutoff for ratio of Huang and Wang indicative of HIE (= 0.644) is shown in broken lines and means in each population by solid lines. The dark boxes and circles represent the babies who developed HIE. HIE developed in babies with the highest value of L:C ratio. The mean L:C ratio in the Thin MSAF group is skewed upwards due to outliers but these are the two babies who develop HIE (at values, a 100 fold of the cut-off of 0.64)

Figure 2 shows distribution of L:C ratios in normal controls and in babies with thin and thick meconium on logarithmic scale. No babies in the control group had L:C ratios of more than 0.64. Five (20%) babies born with thick meconium had levels above 0.64 and 2 of these developed HIE stage II during hospital stay. 11 babies with thin meconium (40%) had levels above the cut-off of 0.64 and among these, HIE developed in the extreme range of figure at L: C ratio of 58.6 and 93.

## Discussion

We have looked at babies who had probable asphyxia long before the time of birth as indicated by thin meconium, against babies asphyxiated around the time of birth, indicated by thick meconium [12,20-22]. We found that the mean L: C ratio in babies with thin meconium was 12 times higher than in those with thick meconium. The two highest values in babies with thin meconium was 93 and 58.6, and in those with thick meconium 1.25 and 1.10. It is significant that it is these four babies (two in each group) who developed convulsions and HIE stage II during hospital stay.

We found that 7% of babies with thin meconium progress to HIE stage II during hospital stay. Similar incidence (6.3%) of severe birth asphyxia (HIE stage II and beyond) in babies born through thin MSAF has been reported earlier28. Although, in our series, we had 2 cases of asphyxia each in babies born through thick and thin MSAF, babies with thick meconium are known to suffer more HIE [9,28,29].

As a priori we have assumed that meconium staining of amnion suggests intrauterine asphyxia. Not all authors are agreed that this is a maker of distress. Some authors consider the presence of meconium as a physiological event [9,30]. Jazayeri et al [20], and Richey et al [21] reported much higher levels of fetal erythopoeitin and Carbonne et al [22] reported fetal anoxia in babies with early passage of meconium. We have shown high levels of L: C ratio among babies with thin meconium. We believe that based on these reports and our findings in this paper, it can be stated that early passage of meconium suggests chronic intrauterine asphyxia.

Thin meconium babies show considerable skewness in their L: C ratio suggesting it to be a heterogeneous group and the ratio a nonspecific marker of asphyxia in this group of newborns. A closer scrutiny (Figure 2) shows at least two groups – one is a compact cluster of babies with L: C ratios comparable to babies with thick meconium and no HIE. The other group of babies with L: C ratios, 10–100 times higher than the mean L: C ratio in the previous cluster and cases of HIE occurred in this extreme group. The first group probably reflects milder-'compensated' asphyxia whereas the latter group probably suffered more severe asphyxia. The latter group can perhaps be better defined when a combination of other antenatal risk factors like FHRT, low apgar score at birth, and thin meconium are used along with L:C ratio.

High L: C ratios in babies with thin meconium need a rational explanation. Lactate is produced by anaerobic metabolism during an asphyxial insult [31-33] and continues to be excreted for long after the insult [34]. Creatinine excretion is dependent upon glomerular filtration and is reduced in asphyxia [35] whereas lactate is cleared partly by hepatic and renal metabolism and partly by tubular secretion [36] using high capacity H+/monocarboxylate cotransporter [37] – operative even in shock [34] and its excretion is reflective of blood levels rather renal status. Babies continue to excrete lactate and creatinine excretion is reduced, the ratios increasing accordingly. Very high lactate in experimental and clinical asphyxia is well known [34,38] and L:C ratios 10–100 times the baseline have been reported previously [26].

The bladder capacity is limited, and repeated voiding occurs in-utero. Once asphyxia occurs, there is continuous excretion of lactate for a long time afterwards. High urine lactate has been seen as much as 48–72 hours after the insult [26], and raised cerebral lactate has been demonstrated up to a month after asphyxia [34]. If the asphyxia occurs early – long before delivery, it may be assumed that all the urine in the bladder consists of post asphyxial urine. If on the other hand, asphyxia occurs just before delivery, the secreted lactate in urine dilutes in the urine already formed in the bladder and, the overall level may be lower. A similar sequence occurs with creatinine and post-asphyxial low-creatinine-urine is diluted in the urine containing normal creatinine levels. In summary high lactate in thin MSAF babies results from continued excretion over long periods and a serial accumulation of lactate.

Thin meconium is a common event in obstetric practice and is generally considered innocuous. We have not looked at the issue of brain damage in this paper and we do not offer any suggestion to the contrary. In our sample of babies with thin meconium, it is those who have L:C ratio 100 times larger than the cut-off of 0.64 who develop HIE. A suitable cutoff of L: C ratio for babies with thin meconium can be evolved by larger studies.

Our study has two important limitations. Firstly, babies were followed only up to discharge from the nursery, and no long-term follow-up for HIE is available. It limits the study from commenting on the issue of brain damage. Secondly we used a 6-hour cutoff for urine collection. It is not unusual for normal babies not to pass urine in up to 24 or even 48 hours. The most severely asphyxiated newborns are oliguric and may not pass urine for even longer. The L:C ratio was not studied in them. Studies on ischemia – reperfusion induced cell damage suggest that the damage is complete by six hours. The "window of opportunity" for rescue medications thus, is only a few hours and in no case more than six hours [39]. Therefore it would perhaps be futile to know about asphyxia outside of this window of six hours, where interventions may not be useful.

In this study we used conventional enzymatic methods to assay lactate and creatinine. Zuppi et al have shown earlier that lactate levels in urine by conventional enzymatic methods are comparable to levels by proton NMR spectroscopy of urine [40]and that two methods can be used interchangeably.

In conclusion, we report that babies with thin meconium normally have much higher L: C ratios and HIE occurs at much higher levels in this group. Larger studies are needed to evolve a cut-off that performs with acceptable sensitivity and specificity in babies with thin meconium.

## Competing interests

The author(s) declare that they have no competing interests.

## Pre-publication history

The pre-publication history for this paper can be accessed here:


